# Development and Validation of Methodology for Estimating Potato Canopy Structure for Field Crop Phenotyping and Improved Breeding

**DOI:** 10.3389/fpls.2021.612843

**Published:** 2021-02-10

**Authors:** Filipe de Jesus Colwell, Jock Souter, Glenn J. Bryan, Lindsey J. Compton, Neil Boonham, Ankush Prashar

**Affiliations:** ^1^School of Natural Environmental Sciences, Newcastle University, Newcastle upon Tyne, United Kingdom; ^2^Survey Solutions Scotland, Edinburgh, United Kingdom; ^3^The James Hutton Institute, Dundee, United Kingdom; ^4^School of Biosciences, University of Birmingham, Birmingham, United Kingdom

**Keywords:** high throughput phenotyping, plant breeding, unmanned aerial vehicles, canopy structure, potato, crop growth and development, crop surface models

## Abstract

Traditional phenotyping techniques have long been a bottleneck in breeding programs and genotype- phenotype association studies in potato, as these methods are labor-intensive and time consuming. In addition, depending on the trait measured and metric adopted, they suffer from varying degrees of user bias and inaccuracy, and hence these challenges have effectively prevented the execution of large-scale population-based field studies. This is true not only for commercial traits (e.g., yield, tuber size, and shape), but also for traits strongly associated with plant performance (e.g., canopy development, canopy architecture, and growth rates). This study demonstrates how the use of point cloud data obtained from low-cost UAV imaging can be used to create 3D surface models of the plant canopy, from which detailed and accurate data on plant height and its distribution, canopy ground cover and canopy volume can be obtained over the growing season. Comparison of the canopy datasets at different temporal points enabled the identification of distinct patterns of canopy development, including different patterns of growth, plant lodging, maturity and senescence. Three varieties are presented as exemplars. Variety Nadine presented the growth pattern of an early maturing variety, showing rapid initial growth followed by rapid onset of senescence and plant death. Varieties Bonnie and Bounty presented the pattern of intermediate to late maturing varieties, with Bonnie also showing early canopy lodging. The methodological approach used in this study may alleviate one of the current bottlenecks in the study of plant development, paving the way for an expansion in the scale of future genotype-phenotype association studies.

## Introduction

Potato (*Solanum tuberosum* L.) is the fourth most important food crop in the world and is regarded as one of the highest yielding crops amongst the staple foods (Birch et al., 2012). In order to feed a growing population under changing climatic conditions, the demand for high yielding and stress tolerant varieties is expected to increase (Birch et al., 2012). While on one hand breeders and biotechnologists have focussed on engineering and breeding crop plants for achieving higher yields and quality, on the other the aim is to maintain agricultural productivity under changing environmental conditions by combating abiotic and biotic stresses.

Breeding programs have traditionally focused on commercially important traits, the major one being yield. Plant performance, along with economic yield, has been shown to be strongly associated with traits related to plant growth and development. These include plant architecture, leaf structure and vascular architecture as some of the major traits that determine overall crop performance (Mathan et al., 2016). Thus, in order to meet the increase in demand for high yielding and stress tolerant crops, it is necessary to expand breeding programs to encompass traits linked to plant growth and development (Prashar et al., 2013; Yang et al., 2017). Engineering developmental and growth traits with the aim of improving plant performance and yield requires a thorough understanding of the underlying genetics, which can be linked with quantitative phenotypic assessments. In the last few decades there has been major developments in genomic and genotyping technologies allowing faster and cheaper creation of complete genetic profiles (Wang et al., 2018), but despite these advances there are few genomics-assisted breeding programs.

One of the major current limiting factors in modern breeding programs is the acquisition of large-scale phenotypic assessment under natural conditions in the field for performance traits. Most of these evaluations have been conducted in controlled environments until very recently (Anithakumari et al., 2011; Khan et al., 2015). However, results from such environments may poorly predict what happens under field conditions (Prashar and Jones, 2014; Williams et al., 2017; Yang et al., 2017). Furthermore, such studies frequently use only a small number of genotypes, and thus may fail to detect Quantitative Trait Loci (QTL) with small effect sizes. In addition, greater precision in phenotypic data collection allows the increase in selection accuracy in breeding, which is a function of heritability, which increases with increased repeatability and thus rate of genetic gain also increases (Araus et al., 2018). Phenotypic evaluation for the genetic study of performance related traits therefore requires large-scale field studies (Prashar et al., 2013; Prashar and Jones, 2014) involving large genetic populations (Furbank and Tester, 2011; Lopes and Reynolds, 2012). However, the requirement for phenotypic assessment is currently one of the major bottlenecks in both genotype-phenotype association studies and large-scale breeding programs.

Current phenotyping methodologies are very laborious and time consuming and therefore impractical for large-scale field studies. In addition, depending on the trait being measured, they can be inaccurate, inconsistent and susceptible to user assessment bias (Friedli et al., 2016; Jimenez-Berni et al., 2018; Wang et al., 2018). For example, leaf area index (LAI) and ground cover (GC) are two traits frequently used in monitoring plant growth (Khurana and McLaren, 1982; Boyd et al., 2002), with most potato yield prediction models requiring at least one of these (Haverkort et al., 2015; Raymundo et al., 2017). Traditional methods such as the use of grids to estimate GC, or light interception based techniques to estimate LAI, are labor-intensive and time consuming (Khurana and McLaren, 1982; Boyd et al., 2002). These challenges limit monitoring to small sample plots, which may not accurately represent the heterogeneity in agricultural fields. This seriously limits the high accuracy and precision that is required in modern agriculture, not only to achieve lower resource inputs and hence environmental impact, but also to accelerate genetic gain through increasing heritability, and hence selection accuracy (Araus et al., 2018). Remote sensing techniques for quantitative assessments and stress detection have been suggested as a possible solution to these limitations (Prashar et al., 2013; Friedli et al., 2016; Yang et al., 2017; Jimenez-Berni et al., 2018; Wang et al., 2018).

Sensing approaches used for crop trait phenotyping and crop monitoring include satellite-based systems, manned aircraft or unmanned aerial vehicle (UAV) linked systems and tractor mounted sensing tools. Satellite remote sensing is capable of monitoring large areas at the same time and has undergone significant improvements in recent years, especially with regards to spatial resolution and increased coverage due to the addition of low orbit satellites. Nevertheless, it still frequently lacks the spatial resolution necessary for precise and detailed canopy phenotyping of relatively small plots. Satellites are also limited to data collection or observations at fixed times, which may not match the phenotyping needs, and by cloud coverage, which may impede data collection during those times (Berni et al., 2009; Matese et al., 2015). In recent decades, UAV technology has become more accurate, and importantly, more affordable. It is capable of monitoring agricultural fields with greater flexibility and higher spatial resolution, in a short time period (Matese et al., 2015; Yang et al., 2017). The nature and extent of the data to be collected with UAVs depends on the type of sensor used (Yang et al., 2017). RGB sensors allow not only visual assessment of the sampled areas, but also the assessment of traits influencing plant development from the point cloud data, such as leaf color, plant height, canopy cover and 3D plant structure. Near-Infrared (NIR) sensors allow estimation of various vegetation indices that can be used to estimate biomass, nitrogen content and disease detection, while thermal sensors are useful for understanding stress and assessing water status (Yang et al., 2017; Zheng et al., 2018; Roitsch et al., 2019). Through the combined use of different types of sensor, numerous traits can be evaluated more efficiently and objectively, with the potential for temporal studies with more frequent data collection points, enabling accurate growth and development models to be created (Prashar et al., 2013; Friedli et al., 2016; Yang et al., 2017; Jimenez-Berni et al., 2018; Wang et al., 2018).

With the images acquired by UAV equipment using various sensors (RGB, multispectral and/or hyperspectral), Structure from Motion (SfM) point cloud data has been used to understand plant growth and development. Early applications of this method include artificial monocultures (e.g., orchards) and diverse biomes (e.g., forestry), which share many of the same challenges, including resource intensive monitoring of large areas and understanding tree crown heterogeneity. For example, a combination of RGB and NIR sensors have been used to develop an object-based image analysis technique for automatically calculating tree height, canopy cover and volume of individual olive trees (Torres-Sánchez et al., 2015), as well as to assess the effect of different pruning methods on olive tree growth (Jiménez-Brenes et al., 2017). UAV based systems have also shown potential for estimating flower biodiversity (Getzin et al., 2012) and for the creation of a rapid and accurate forest census (Mohan et al., 2017). However, applications in agriculture have mostly been limited to cereals (Bendig et al., 2013, 2014, 2015; Holman et al., 2016; Jin et al., 2017) and cotton (Xu et al., 2019). In potato, UAV acquired images have been used to estimate plant emergence (Sankaran et al., 2017; Li et al., 2019) and assess disease severity (Sugiura et al., 2016; Franceschini et al., 2017). However, there have been no published studies related to plant structure, including canopy architecture and development, under field conditions.

This article evaluates the use of a low-cost UAV system, mainly in the form of RGB imaging resources and obtained datasets, for understanding plant growth and development in potato under natural field conditions. The image datasets from this system are used to develop new methodology for quantifying canopy growth parameters and assessing canopy variability through developing crop growth and development models, with validation using ground truth datasets. This methodology enables quantitative trait assessment and modeling of growth and development parameters in potato, which can allow high-throughput phenotyping of canopy traits for integration with large-scale genetic datasets and hence the improvement of future potato breeding programs.

## Materials and Methods

### Plant Material and Field Layout

The data used in this paper forms part of a large study that was performed at Nafferton Farm, Newcastle University, United Kingdom, with field trials at 54°59′12.0″N 1°53′33.9″W/54.986655, −1.892751 and 54°58′51.3″N 1°53′56.5″W/54.980924, −1.899018, in 2017 and 2018 respectively. A total of 297 varieties of potato (*Solanum tuberosum* L.), which form a large part of a tetraploid variety association panel available at The James Hutton Institute (Sharma et al., 2018), were planted in April 2017 and May 2018. The experimental design consisted of two replicate blocks for each of two management systems (organic and conventional), making a total of 4 blocks. Each block consisted of 6 rows spaced 90 cm apart and comprising 50 plots per row. Each plot contained 3 plants of a given variety planted 35 cm apart. Spacing of 90 cm was maintained between plots within each row. To minimize edge effects, a row of guard plants was planted surrounding each block. Both conventional and organic trials were conducted using their respective standard management practices.

### UAV Flight Parameters

UAV flights were performed in collaboration with Survey Solutions Scotland using a fixed wing UX5 HP UAV (Trimble, Sunnyvale, California, United States). The UX5 HP uses Global Navigation Satellite System (GNSS) post-processed kinematic techniques to determine the UAV trajectory. Images were taken using a Sony α7R 36MP full frame 35 mm RGB camera with a custom made Voigtlander 35 mm lens. The 35 mm lens was selected to deliver a 1.0 cm Ground Sample Distance (GSD) at 75 m Above Ground Level (AGL), while also offering pixel sizes down to 4.9 μm, to maximize the signal to noise ratio and dynamic range, while maintaining affordability. Given the importance of the canopy volume in this research, a UAV sensor with a global rather than a sliding shutter was selected for the imagery as this greatly reduces noise in the images, which leads to a much cleaner and more precise deliverable. The data was collected at 75 m altitude with overlaps of 85% (both front and side) between neighboring images. The speed of flight was nominally 85 km/h, therefore flying height was restricted to 75 m AGL to minimize image distortion due to motion blur. Details of flight dates and their relation to canopy development in days after planting are given in Table 1.

**TABLE 1:** Planting and UAV flight schedule for assessing potato canopy characteristics.

Year	Planting	Flight date	Days after planting
2017	28-04-2017	07-07-2017	70
		24-07-2017	87
		08-08-2017	102
2018	03-05-2018	05-06-2018	33
		04-07-2018	62
		06-08-2018	95
		28-08-2018	117

### Image and Data Analysis

The images acquired using UAV were processed and analyzed using the Trimble Business Center (TBC) software version 4.1 and 5.0, for the 2017 and 2018 datasets, respectively (Trimble, Sunnyvale, California, United States). This includes the subsequent use of GCP referencing, point cloud data generation, creation of digital surface models, manual plot demarcation, computation of difference models and canopy data acquisition e.g., canopy cover and volume (further details in the following sections). Plant height data clean up, subsequent statistical analysis (regression and correlation) and other data processing was carried out in R (R: Project for Statistical Computing, The R foundation) using the following packages: dplyr, ggplot2, gridExtra, Hmisc, plotrix, plyr, SDMtools, tidyr, tidyverse (Lemon, 2006; Wickham, 2011, 2016; Vanderwal et al., 2014; Wickham et al., 2015, 2019; Baptiste, 2017; Wickham and Henry, 2019; Harrell, 2020).

## UAV Data Pre-Processing

### UAV Trajectory Processing

Raw GNSS data was recorded in the UX5 HP UAV by the on-board 336-channel multi constellation GNSS receiver, which is downloaded at the end of the flight and processed against a local base station situated within our flight area. The local base position was established by processing the local base against Ordnance Survey CORS (Continuously Operating Reference Stations), sourcing 1 hourly RINEX data, which provide GNSS data at reference stations coordinated in ETRS89 (ETRF2009.756). Processing the local base station relative to known Ordnance Survey CORS (OSNet) helps in establishing the position of the local base for each UAV flight and its repeatability is assured. Processing multi constellation GNSS data relative to fixed OS CORS typically gave estimated precisions of ≈5 mm in plan and ≈20 mm in height (at 95% confidence) over 30 km baselines. OSTN15 CORS stations, relative to each other, are considered error free. The processed base stations used the OSTN15 transformation model and OSGM15 geoid correction surface to convert the ETRS89 global WGS84 coordinates into Ordnance Survey (OS) grid coordinates. The local base was processed (using the final local base station coordinate) against the 20 Hz UAV data to produce a continuous flight trajectory of the UAV. This estimated *a posteriori* trajectory accuracy of 97.20% @ 0–5 cm and 2.60% @ 5–15 cm, and the remaining values were considered outliers.

PPK (Post-processed Kinematic) was used to create the trajectory as it is more robust than alternative methods, which may rely on radio or other communications. In addition, precise ephemerides can be incorporated into the processing later, to enhance the baseline processing algorithms if needed. Although we used a processed UAV trajectory, we still used and placed Ground Control Points (GCPs) as required, but the number of these can be greatly reduced in comparison to non-PPK methods.

### Photogrammetric Processing

The UAV trajectory was processed in TBC software, with feedback events recorded at better than millisecond accuracy. This helps to precisely establish the location of the photo center of each image at the time of exposure. Having image positions at the cm level negates the need for dense pixel matching, a process which is required in non-PPK aerial photogrammetric processing. It also greatly reduces the need for intensive, time consuming and expensive GCP placement, which would be impractical given the expected development of the canopy in this research.

Around 6 GCPs were placed in the periphery of the trial and were used in all flights to ensure repeatable and accurate deliverables and to generate an accurate camera calibration. In addition to being measured as vectors from the local base, the GCPs were also georeferenced using Network RTK (Real-time Kinematic), which provides an independent check on the GCP coordinates. These GCP coordinates were fixed for the duration of the project, thus providing a common datum for all flights in a given year.

### Image Processing

The images were imported into TBC software at the same time as the Raw GNSS data, so that when the trajectory is processed and the event markers created, each image will be positioned in the correct 3D position. To resolve the orientation of each image, i.e., the omega, phi and kappa rotations, a precise Interior Orientation (IO) is computed using the GNSS positions, i.e., a tie point adjustment, highlighting how well the images tie together. In non-PPK processing, this is a computer intensive and time-consuming process. However, a PPK trajectory resolves for the image location, thus only the orientation needs to be resolved, resulting in a more rapid and robust solution.

The IO was followed by an exterior orientation (EO) with camera calibration. For the EO, visible GCPs in each image were “picked” so that the real-world coordinates are allocated to the GCP image coordinates (as produced by RTK observations and verified by Network RTK). As mentioned above, GCPs allow camera calibration and computation of distortion parameters for the lens. The combination of GNSS and GCPs also allows computation of the focal length. Both of these parameters are a necessity for creating “noiseless” deliverables. After the EO is performed, the flight report is analyzed for errors and accuracy. The low errors and high confidence accuracy confirms the validity of the flight and ensures observation repeatability over the duration of the experiment (data not presented).

## UAV Data Processing

### Deliverable Creation

The next step after measuring accuracy and acceptance of the EO results is to create deliverables (e.g., point cloud, orthophoto, etc.). Both point cloud and true orthophotos require well-orientated images. Different types of surfaces can be generated from orthomosaic images. Surface generation is the creation of a point cloud and it requires at least two, and preferably more, overlapping images. Insufficient overlap produces noise, or worse, gaps in the data. A surface was generated using the maximum resolution available (appropriate to the flight parameters) using a Cost-based Matching algorithm. Briefly, the algorithm uses pixel-by-pixel matching, rather than an area based or feature based technique, though a detailed discussion of the algorithm is beyond the scope of this work.

The orthophoto (i.e., geometrically corrected or orthorectified) was created after processing of the point cloud surface, to give an image where the scale is uniform and true. A “True” orthophoto rather than “Classic orthophoto” was selected because it uses the surface model to calculate occlusions and fill them in from other images, which is essential when the canopy is not uniform. A “Classic” orthophoto, on the other hand, would require bare Earth.

### Preliminary Data Clean Up

**FIGURE 1:**
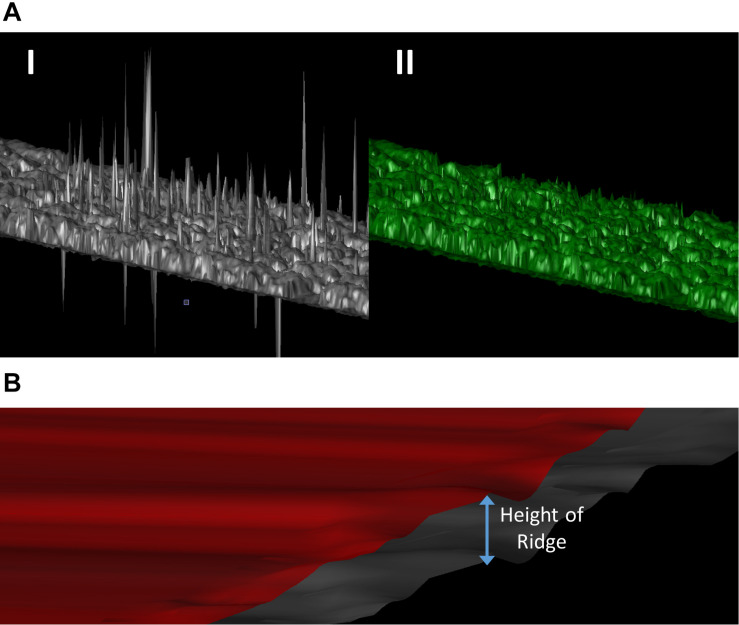
**(A)** 3D representation of the point cloud before (I) and after (II) the manual removal of erroneous points. **(B)** Generation of a soil reference surface (red surface) by increasing every point of the topographical surface (gray surface) by the average height of all the ridges in the block.

Raw data and the generated point clouds typically include errors due to several factors. These include vegetation movement due to wind, UAV crabbing in flight, which lessens the expected image overlap and bad reflection points (i.e., noise due to inadequate overlap and image uncertainty). These erroneous points need to be removed from the point cloud (Figure 1A). This was accomplished by the manual removal of points that would be considered impossible, as determined by their height based on proximity to other points, position in the field, and visual inspection of the point cloud. We used a manual removal process because the outlier points in our data were sparse and inconsistent in local point density. Automatic outlier removal methods are available if needed, such as the discontinuous operators-based method (Ning et al., 2018).

### Surface Model Creation

Following clean up, the point cloud data was used to generate two surfaces (where surface is referred here as 3D models generated from point cloud data), including one for the soil reference and another for the canopy. Surfaces created in the TBC software are a 3D digital representation of topography (in this case, canopy), formed by a mesh of contiguous triangles, and sometimes referred to as a Triangulated Irregular Network (TIN). The triangles are connected at their vertices, which are defined by points with horizontal positions (*X* and *Y*–values) and elevations (*Z*-values), i.e., points in a point cloud forming three sided planar faces. The surface model from a point cloud is a simple set of triangles, but can be enhanced by the inclusion (or omission) of boundaries, break lines, and points, etc. that make up the surface model and that are used to define its shape.

Canopy surfaces in our case used the totality of the point cloud within each experimental block. Ideally, the creation of a soil reference surface would use point cloud data from below the canopy, but Structure from Motion imagery does not permit this, especially when plants are growing. Therefore, to overcome this limitation a surface was created to estimate the soil topography. Since potatoes are grown on ridges, a topographical surface was first created using the bare soil surrounding the plants, hence excluding the ridges and any potential vegetation. Second, the height of every point on this surface was increased by the average height of all the ridges in the block to construct a raised surface (Figure 1B).

### Difference Model Creation and Plot Demarcation

**FIGURE 2:**
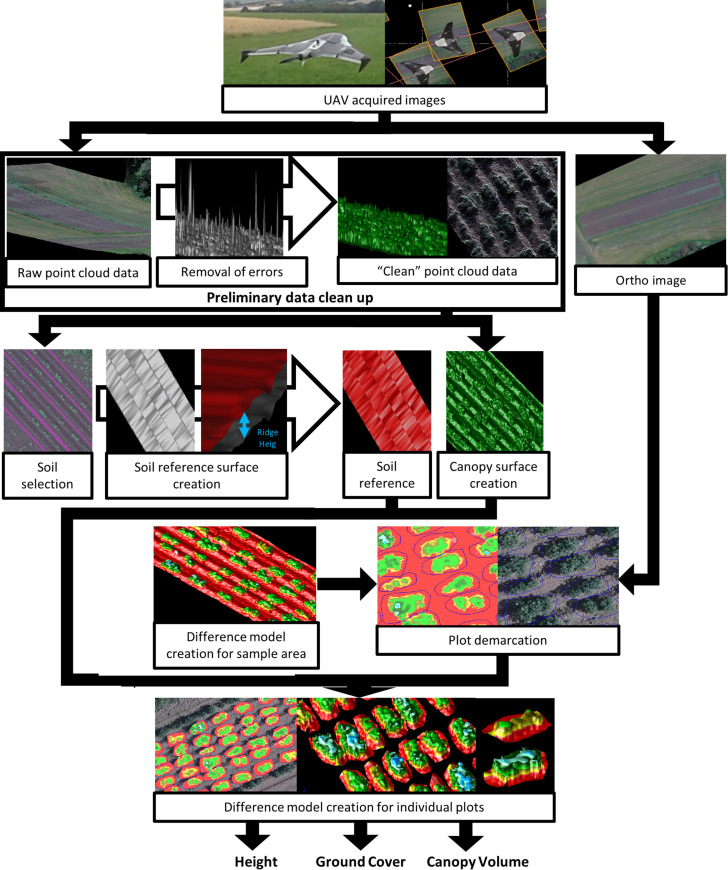
Methodological flowchart showing the acquisition of quantitative data on potato plant canopy structure (e.g., canopy volume, canopy ground cover, and plant height) using a Structure from Motion algorithm from UAV acquired images.

As a next step after generating surfaces, difference models were created for the entirety of each experimental block. Difference models are a 3D representation of a model, where each point in the model has the elevation difference between two surfaces on the same point. Once generated, this difference model was then used in combination with the orthomosaic image to accurately demarcate individual plots. The demarcation of individual plots made it possible to create difference models for each and every plot in each block, using the same surfaces that were created previously, from which we are able to extract canopy volume, ground cover and canopy height datasets for further analysis. Details of the workflow are shown in Figure 2.

### Difference Model Computation Methods

**FIGURE 3:**
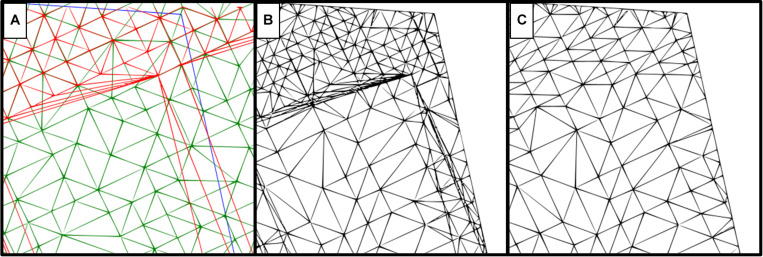
Comparison of two difference model computation methods using the same reference surfaces. **(A)** Surfaces used for computation, where red represents the soil reference, green is the canopy surface generated from the point cloud, and a blue line demarcates the plot border. **(B)** Difference model using the “trace all triangles” method. **(C)** Difference model using the “do not track breaklines” method.

Two difference model computation methods were compared (Figure 3). The “trace all triangles” method (Figure 3B) creates a new vertex at each point of the point cloud where soil surface and canopy surface triangles either overlap or intersect, while taking into account any existing breaklines created during demarcation. These vertices serve as new points for the creation of the difference model; therefore, the resulting difference model has a denser mesh of vertices than the original surfaces. The “do not track breaklines” method (Figure 3C) uses only existing points of the point cloud to create a difference model, ignoring breaklines and not creating new vertices, with the generated difference model having the same density of vertexes as the original surfaces. For comparison, 300 field plots were selected and both methods were applied to generate the triangular mesh. Subsequent calculations of plot volume were compared using Spearman’s rank correlation coefficient.

### Plant Height Calculation

The difference model helps us build up the height data at any point within the plot area, at a resolution of 1 cm. This allows us not only to determine the highest point within the demarcated area (representing the maximum height of the 3 plants within the plot), but also allows the construction of a frequency table of the height distribution that provides information on the canopy structure and allows calculation of the average height of the plants.

### Plant Height Data Clean Up

Preliminary point cloud clean-up removes the more noticeable errors, which tend to be the impossible or unrealistic values. However, smaller errors tend to remain in the point cloud. These smaller errors have a negligible effect on the average height, but maximum height is more susceptible to influence. Weeds are also a probable source of errors when it is not possible to manually remove them from the potato plants. An integrated approach using different imaging sensors (not illustrated here) is more valid in this scenario but falls outside the scope of this work. Small weeds will provide a small, potentially negligible, effect on the histograms and the average height. However, maximum height can easily be overestimated because of a single weed plant that outgrows the potato plants, thus necessitating a more precise clean up. First, all points above 1.2 m were removed, as potato plants do not reach this height. Second, further clean-up was achieved by using the standard deviation (SD) of the plot canopy height distribution as a cut-off for the maximum height. Cut-offs of 2, 2.5, and 3 SDs above the mean were evaluated by comparing post cut-off data with field/proximal data using regression analysis and other graphical visualizations including histograms to observe the effects on individual plots.

### Canopy Ground Cover and Canopy Volume

Canopy ground cover and canopy volume are defined as the sum planimetric area and total canopy volume, respectively, that is above the level of the soil reference surface in the difference model for each plot.

## Proximal Data Acquisition

Ground truth data was collected for comparison with measurements obtained from the UAV point cloud data approach.

Plant height was measured proximally using a ruler in randomly selected plots on the same day as UAV data collection. The highest contact point of the plant for each of the 3 plants in each plot was recorded. The maximum height from the 3 plants per plot was compared to the maximum height determined from the UAV data.

Leaf Area Index (LAI) data was obtained using a ceptometer (ACCUPAR LP-80, METER ENVIRONMENT, part of METER Group, Inc. United States). The ratio of the length of the horizontal to the vertical axis of the spheroid described by the leaf angle distribution of a canopy was assumed to be 2 for the leaf distribution parameter in potato plants. The sensor was angled so that the angle to the ridge was kept the same and would cover all plants within a plot (Supplementary Figure S1). All LAI measurements were taken in tandem with the field height measurements in 2018.

## Results

### Ground Truth Versus Image Based Plant Height Measurements

**FIGURE 4:**
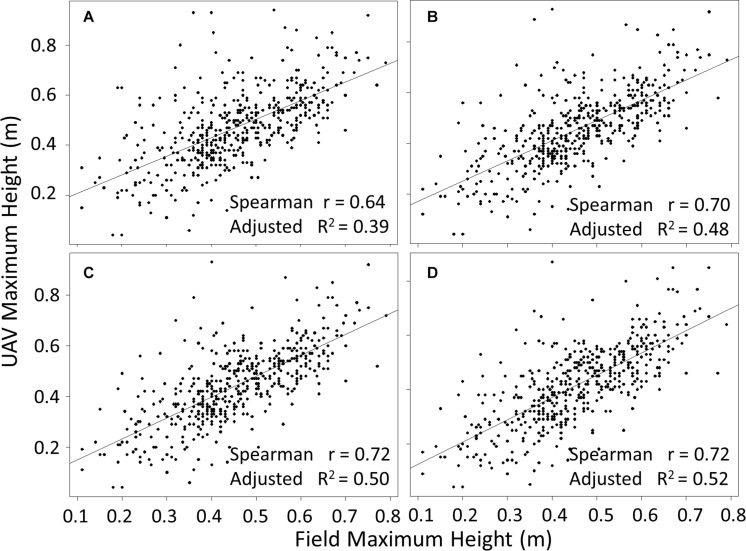
Comparison of maximum height computed from UAV flights with proximal ground truth measurements at plot level, for pre clean upUAV data **(A)**, and data cleaned using cut-offs of 3 **(B)**, 2.5 **(C)**, and 2 **(D)** standard deviations above the mean. *p* < 0.01 in all cases and *n* = 488.

**FIGURE 5:**
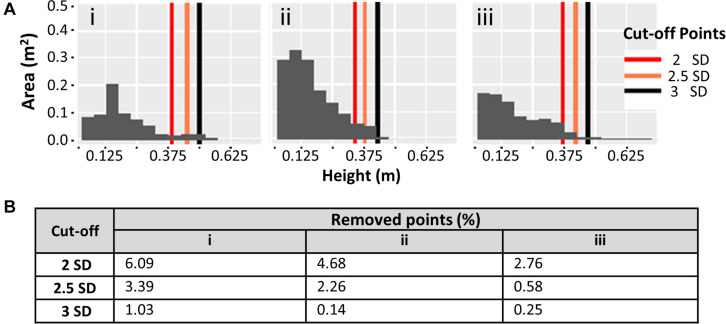
**(A)** Effect of 3 different standard deviation (SD) cut-offs on plot canopy datasets. Histograms represent the canopy area coverage at different height levels using pre clean up canopy height data. **(i–iii)** Demonstrate 3 exemplar canopy plots. **(B)** Percentage of points removed from the point cloud at each SD cut-off for the corresponding histograms in **(A)**.

Plant height measurements from field collected proximal data were compared with measurements based on UAV imaging. A two-step data cleaning procedure for the UAV image data involved manual removal of obvious outliers to produce “pre clean up” data, followed by a second round of cleaning using various standard deviation cut-offs based on the plot height dataset (see section “Materials and Methods”). Data cleaning significantly increased the concordance between field and UAV data (Figure 4). The pre clean up data showed a relatively low *R*^2^ of 0.39 (*p* < 0.01), and this value increased to moderate levels for cut-offs of 3 SD, 2.5 SD and 2 SD (*R*^2^ of 0.48, 0.50, and 0.52, respectively, all *p* < 0.01). Observations from Figure 4 highlight that the different cut-off levels are not significantly different (data not presented). To determine the most appropriate cut-off level, we visually evaluated the risk of removing real canopy data in three selected exemplar plots (representing relatively common height distribution profiles) (Figure 5). The first example plot in Figure 5Ai presents an ideal situation in which all cut-offs remove only the elongated tail that is caused by computational errors and weeds integrated within the canopy structure of the plot, thus affecting the maximum height measurements. In the other two exemplar plots shown in Figures 5Aii,iii, the 2 SD cut-off point clearly removes part of the canopy, while the 2.5 SD cut-off removes part of the canopy in Figure 5Aii, but not in Figure 5Aiii. This increase in percentage of points removed is also exhibited in Figure 5B. The combined analysis from Figures 4, 5 suggested that the 3 SD cut-off was the most appropriate as it deterred the removal of the canopy data while also removing most of the noise.

**FIGURE 6:**
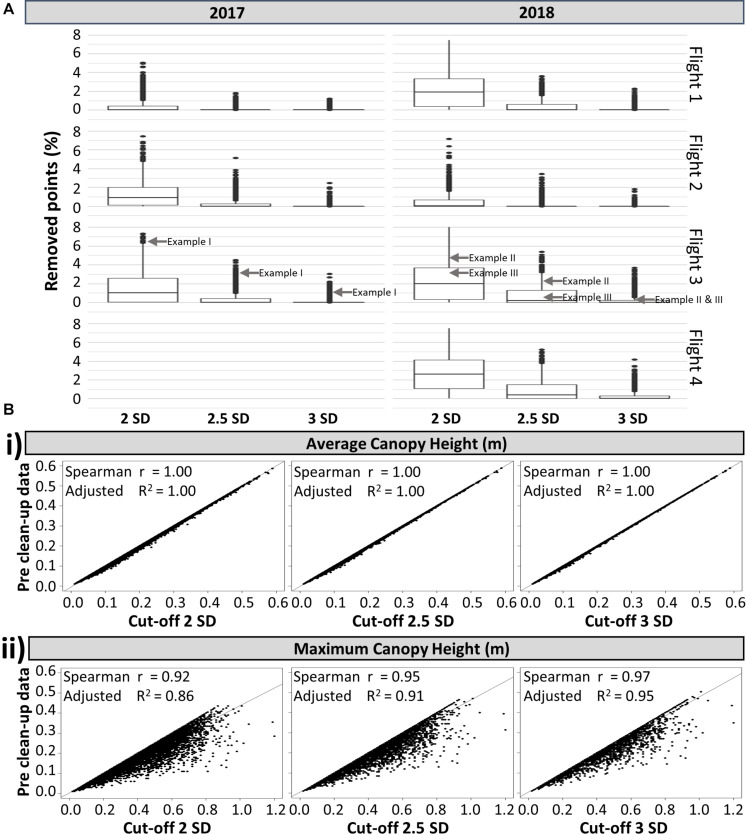
**(A)** Box-plot of the percentage of points removed from the point cloud of individual plots based on 3 different standard deviation (SD) cut-offs, shown by flight and by year (*n* = 8,264). The locations of the exemplar plots shown in Figure 5 are indicated with gray arrows. **(B)** Relationship between the pre clean up dataset and post clean up dataset using three SD cut-offs, for the: **(i)** average canopy height and **(ii)** maximum canopy height. All analyses have a *p* < 0.01, *n* = 8,264.

To gain a clearer understanding of how the observations made in Figure 5 were reflected in the rest of the field, Figure 6A presents the overall effects of the same standard deviation cut-offs on all experimental plots (individual histograms are not shown). In accordance with Figure 5, the 2 SD cut-off removed the highest percentage of data points. A large number of plots had relatively high percentages of data removed, with an overall average of more than 1%, indicating that canopy data is removed from most plots. The 2.5 SD cut-off performed better, but still removed the canopy data from a significant portion of the experimental plots. As expected, the 3 SD cut-off removed the lowest percentage of data points, but still preserved the canopy profile of all the plots in the experiment (a similar scenario to the one shown in Figure 5Ai, where over 1% of points are removed from a particularly pronounced elongated tail).

Independent of the nature of the data points (i.e. canopy data versus errors), their removal will always reduce the measured average and maximum canopy heights (Figures 6Bi,ii, respectively). As expected, the estimated maximum canopy height based on the 2 SD cut-off showed the most divergence from the estimate based on pre clean up data, while the 2.5 and 3 SD cut-offs led to a stronger correlation (Figure 6Bii). In contrast, for the average canopy height, the three different standard deviation cut-offs have no effect (Figure 6Bi). This indicated that the average height was not significantly affected by the existence of elongated tails caused by computational errors and weeds integrated within the canopy structure. Similarly, comparing average height with proximally measured field maximum height, there was no discernible difference between using pre clean up data or data cleaned using different SD cut-offs, with all comparisons showing an adjusted *R*^2^ of 0.46 and all *p* < 0.01. Thus, the point cloud generated average height provided a more consistent measure than the maximum height for evaluating canopy height in potato.

### Comparison of Difference Model Computation Methods

There are various methods for constructing difference models in the TBC software. These differ according to feature usage, including breaklines and newly extrapolated points where surfaces intersect, in addition to existing points in the surfaces. We compared the “trace all triangles” method with the “do not track breaklines” method and evaluated the pros and cons of each.

The “trace all triangles” method (Figure 3B) had a much denser mesh generation compared with the “do not track breaklines” method (Figure 3C), especially where the canopy and soil references overlapped. To assess any potential impact on our results, 300 plots were analyzed using both computational methods. There was no significant difference between the two methods for the canopy volume generated (Spearman’s rank correlation *r* = 1.00, adjusted *R*^2^ = 1.00, *p* < 0.01, *n* = 300; Supplementary Figure S2). However, the “trace all triangles” method was computationally demanding and took significantly longer (48 h) than the “do not track breaklines” method (25 min) to compute all 300 plots. Though it was not based on a quantitative in-depth analysis when compared to the “trace all triangles” method, the “do not track breaklines” method therefore seemed the most appropriate based on the required computational resources.

### Relationship of Canopy Traits With LAI

**FIGURE 7:**
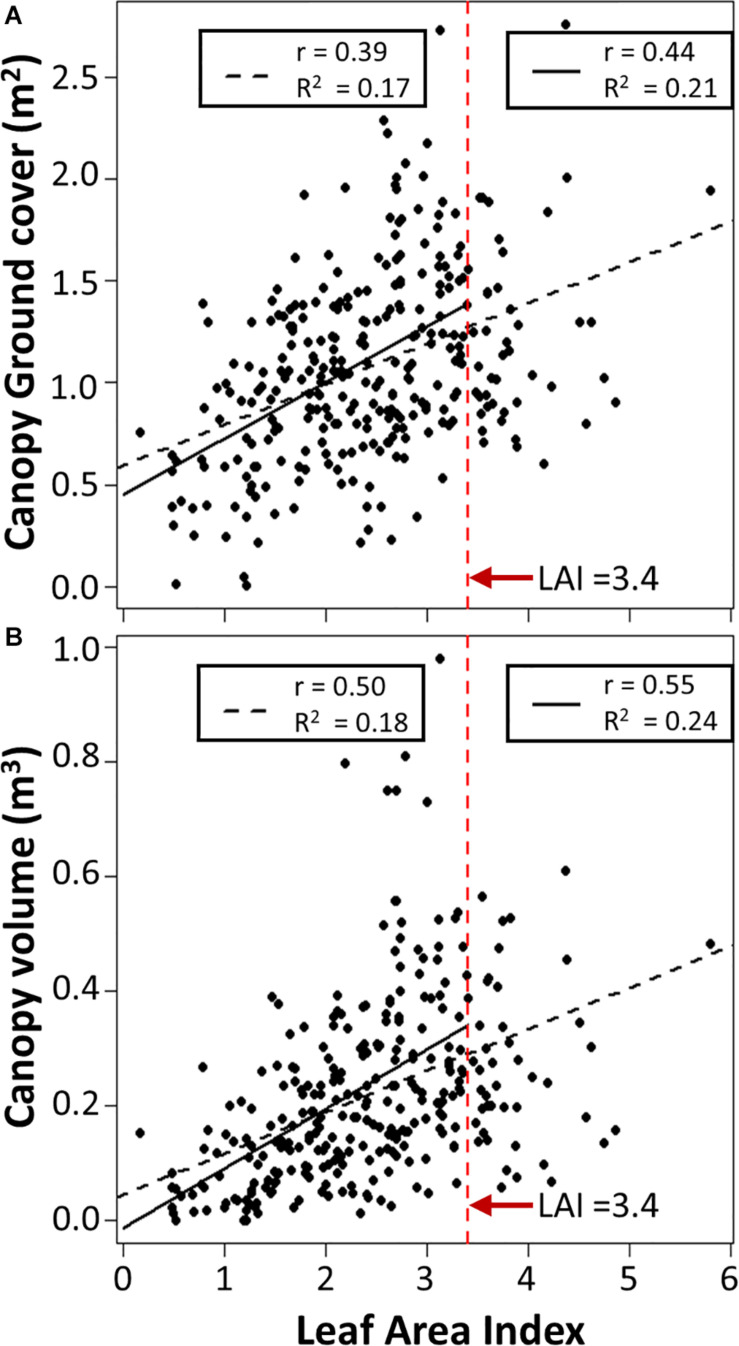
Relationship between Leaf Area Index and computed **(A)** Canopy ground cover and **(B)** Canopy volume for all observations (dashed black line, *n* = 291) or when observations with LAI above 3.4 are discarded (solid black line, *n* = 251). r is the Spearman’s rank correlation, R^2^ is the adjusted R^2^. All statistical tests have a *p* < 0.01.

Previous potato studies (Haverkort et al., 1991; Boyd et al., 2002) indicated that when leaf area index (LAI) is higher than 3, there is no longer a relationship between LAI and ground cover, because the canopy has grown to the point of achieving full ground cover. We found LAI correlated significantly (*p* < 0.01) with both canopy volume and canopy ground cover, with correlation coefficients of 0.50 and 0.39, respectively (Figure 7). The strongest relationships with canopy volume (*r* = 0.55, *p* < 0.01) and ground cover (*r* = 0.44, *p* < 0.01) were identified when observations with LAI above 3.4 were discarded, as those discarded observations showed no relationship with either canopy trait.

### Temporal Variation of Canopy Characteristics

The combination of canopy height, ground cover and volume can provide comprehensive canopy size information. Though average height is a more robust parameter than maximum height for canopy height measurement, it does not provide quantitative information on canopy shape or structure. Therefore, height distribution data is important for characterizing the canopy profile. The combined information on the canopy size and shape from sequential flights helps to better understand the pattern of canopy growth and development and the current stage of plant growth.

**FIGURE 8:**
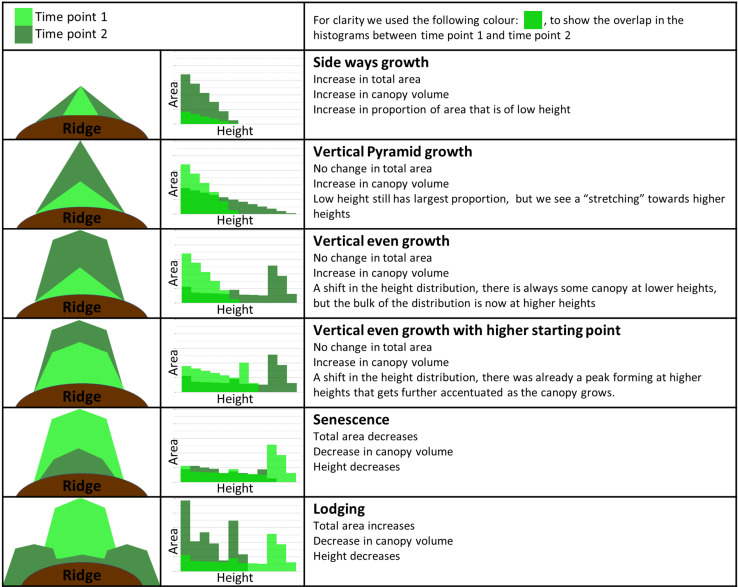
Guide to interpretation of canopy development data at two time points. Column 1 shows a diagrammatic representation of the canopy side profile with the corresponding height distribution histograms located in column 2 (simulated data). Time points 1 and 2 are shown in light green and dark green, respectively. Column 3 defines the name given to each growth pattern, followed by a brief description of the most relevant changes between the two time points.

Figure 8 presents a simplified version of the more complex real-world plant growth pattern data. It provides a general guide for the interpretation of growth patterns over time and illustrates how UAV data can be used to infer canopy development and size distribution. This guide can be a useful tool not only for monitoring individual plant/canopy development, but also to understand varietal variation. The canopy exemplars in Figure 8 show several growth patterns, canopy shapes and their corresponding height distribution histograms. In the simulated growth pattern, we mimic the increase in area/ground cover (e.g., “sideways growth”), the increase in height (e.g., “vertical pyramid growth,” “vertical even growth with higher starting point”), and present examples of plant lodging and plant senescence.

**FIGURE 9:**
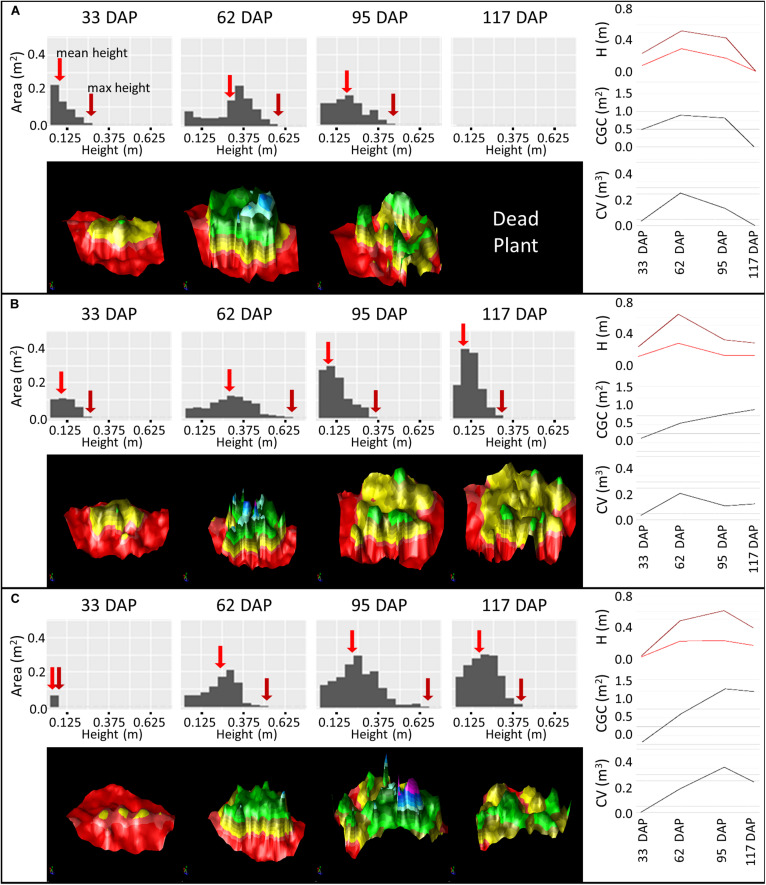
Monitoring Canopy Volume (CV), Canopy Ground Cover (CGC) and Plant Height (H) over the growing season using difference models of three varieties: Nadine **(A)**, Bonnie **(B)** and Bounty **(C)** from 33 to 117 days after planting (DAP). Histograms represent the height distribution of the corresponding difference model. Maximum height is indicated with a dark red arrow and mean height with a medium red arrow. The difference model is color coded based on height, with red (<0 cm, soil), yellow (0–20 cm), green (20–40 cm), blue (40–60 cm), and purple (>60 cm).

We chose three varieties to provide an illustration of not only how the interpretation of canopy data can be used to infer canopy development, but also how data gathered over the entire growing season allows the determination of maturity (Figure 9). In variety Nadine (Figure 9A), we observed that from 33 days after planting (DAP) to 62 DAP the change in height distribution showed an almost perfect example of the “Vertical even growth” pattern (Figure 8), with the canopy changing from a pyramid like shape to a more bulky rectangular shape. This growth was associated with an increase in canopy volume, ground cover and height. By 95 DAP, senescence had begun, with the accompanying decrease in canopy volume, ground cover and height, as expected. The height distribution also resembled the “senescence” pattern (Figure 8), and by 117 DAP the canopy had already senesced completely. A quick early growth followed by rapid senescence indicated that Nadine was an early maturing variety.

In variety Bonnie (Figure 9B), we observed that from 33 DAP to 62 DAP there was an expected increase in canopy height, ground cover and volume. However, unlike Nadine, the height distribution pattern was not perfectly matched to the one shown in the guide (Figure 8). At both 33 and 62 DAP, the canopy growth seemed to be occurring more like “vertical even growth,” but the height in the middle part of the canopy increased more rapidly than the remaining area, i.e., the height of the main stem seemed to be increasing more quickly relative to the side stems. At 95 DAP, we observed a good example of plant lodging, with the bulk of the canopy shifting toward lower height, and canopy ground cover continuing to increase despite the decrease in canopy volume and height. This variety also serves as an example of continued growth after lodging, as we observed an increase in canopy volume, ground cover and height at 117 DAP, which was also evident in the corresponding 3D model, with the growth of new stems at the center of the canopy (Figure 9B). The continuous growth until 117 DAP indicated that this variety was an intermediate to late maturing variety, but that lodging probably hinders its full growth potential.

Variety Bounty (Figure 9C) had a small canopy at 33 DAP due to late emergence. By 62 DAP it presented a similar height distribution pattern to Nadine but was clearly smaller. At 95 DAP the canopy size continued to increase. When looking at the height distribution pattern, there was a noticeable increase in area in the low to medium height range, with little increase in the maximum height. Combined with the consistent increase in the ground cover growth from 33 to 95 DAP, this suggested that either there was a chance of small partial lodging, which allowed the plant to increase its ground cover, or that this variety invested more in lateral growth than vertical growth (for more photosynthetic capacity). Only at 117 DAP was the start of senescence observed. This pattern of continuous growth until nearly the end of the season indicated that this variety was either intermediate leaning toward late maturing, or a late maturing variety.

## Discussion

### Approaches for Determining Soil Topography

One of the major difficulties in using Structure from Motion to generate point clouds is the inability to determine the topography of the soil below the plant canopy. To overcome this hurdle, a soil surface was created using soil surrounding the plots and then a compensation was made for the average ridge height. This is the first study to implement and evaluate this new method in ridged crops, which is an extrapolation of the method commonly used in height assessment studies (Bendig et al., 2015; Holman et al., 2016; Mohan et al., 2017; Hassan et al., 2019). This method depends on the existence of easily identifiable bare soil in close proximity to the crops, which is easy at the beginning of the season (also evident from Holman et al., 2016; Mohan et al., 2017), but may become impossible when the plant canopy achieves total ground coverage, depending on canopy structure.

An alternative method would be to perform a UAV flight before plant emergence begins and use the soil topography as a reference for all subsequent data collection points. This method has the dual advantage of capturing the true ridge height and allowing plant growth to be monitored below the ridge height, which is particularly relevant later in the season when lodging and plant senescence significantly alter the canopy structure. However, in some potato planting cases (e.g., in organic systems), re-ridging is important, which further motivated the evaluation of the new technique in this work. Both methods share the assumption that soil topography remains relatively constant throughout the growing season. However, changes in soil topography during the season could be a significant factor during data collection for crop phenotyping and growth monitoring studies. Such effects are expected to be more significant when plots are used for scientific research, and hence subject to higher intervention rates, but may be much less in large commercial agricultural fields. Simultaneous use of both methods may be beneficial, as the measurement of canopy proportion that is above or below the ridge height may be of use in monitoring senescence and plant lodging.

### Consistency Between Proximal and UAV Based Measurements

Previous studies in various crops including rice, wheat and maize, have demonstrated high correlations (*r* > 0.90) between remote and proximal measurements of plant height (Bendig et al., 2015; Holman et al., 2016; Li et al., 2016). Here, we observed correlations between 0.64 and 0.7 (*p* < 0.01) between plant height measured proximally using a ruler in the field and height measured using point cloud UAV data after clean up. The comparatively low level of correlation we observed may be attributed to several factors. One important difference between potato and the crops in previous studies is the canopy architecture. Potato plants are usually grown from tuber seeds, which result in a potato plant canopy composed of several main stems (Struik, 2007) in the form of a shrub, while the previously studied crops (mostly cereals) either have only one main stem or tend to have mostly vertical growth. This increases user bias error in the field measurement of potato plant height, as the user may erroneously measure a stem that is not the same as the one selected with point cloud data. This error may be removed by using a GPS based height measurement tool to ensure that the same point of proximal measurement in the field is compared with its UAV cloud dataset counterpart. The potato canopy is also shorter than some previous crops analyzed, and hence the relatively fixed error associated with Structure from Motion point clouds can have a slightly greater proportional impact on measurement. The spatial resolution or flight conditions also play an important role in the calculation of height, as lower flight altitudes generate more accurate height estimates (Holman et al., 2016). This may well have played a role in our datasets as we used fixed wing aircraft with most flight data collected at around 75 m with over 85% overlap in data collection. A multi-copter would allow more freedom regarding control over the spatial resolution, with similar image overlaps.

### Height Measurements

Maximum height is commonly used to represent plant growth characteristics in shrub plants including potato, cotton, and fruit trees, and in cereals where data is collected from large plots and maximum height based on a small number of point clouds is used. We have shown that compared with the maximum height, the average height provides a more consistent measure of plant height, which is both more robust to the data cleaning strategy, and better representative of the entire canopy height distribution, as demonstrated using temporal data on the canopy structure of three varieties (Figure 9). In addition, the use of maximum height data has higher potential for user bias during in-field measurements, and for computational error effects while analyzing the UAV point cloud data. Thus, though it is almost impossible to verify using traditional in-field measurements, we recommend the use of average height as it gives a much better representation of plant growth, which is of utmost importance when attempting to understand the genotype-phenotype relationship in plant breeding. The improved precision in phenotype datasets allow us to decrease the error values and hence provides the opportunity to improve the heritability of traits (Cobb et al., 2013). Thus, the accuracy and precision in phenotyping provide the necessary tools to empower the next generation of linkage mapping and association studies and further improve the results of genomic selection (Cobb et al., 2013; Prashar and Jones, 2014; Bhat et al., 2016; Melandri et al., 2019).

### Canopy Traits and LAI

Previous studies have reported correlations between leaf area index and ground cover ranging from 0.52 to 0.92 based on analyses of one or two potato varieties (Haverkort et al., 1991; Boyd et al., 2002). These studies also highlight high correlation with canopy cover for LAIvalues below 3, but no relationship for LAI above 3, due to complete ground cover. Here, we observed a lower correlation of *r* = 0.44 (*p* < 0.01) and a similar cut-off point of LAI 3.4 was established. The reduced correlation within our dataset is likely due to the high levels of varietal variation in canopy architecture compared to previous studies where analysis was carried out on one or two varieties. Canopy volume exhibits a higher correlation with LAI (*r* = 0.55, *p* < 0.01) in our data, because even though UAV measured canopy volume does not consider canopy leaf density, larger canopies are more likely to have a higher leaf density and hence higher LAI. Further developments for UAV determined canopy volume, ground cover and LAI would have to take into consideration varietal data to enable integration into potato yield prediction models in the future.

### Plant Growth and Development Monitoring

Crop monitoring for growth and performance during development is an important aspect of agricultural management, and not only allows creation of yield prediction models, but also enables implementation of timely interventions to ensure optimal yields. Therefore, while individual flights provide useful point information on the size and the general canopy health of the plants, it is the continuous data integration of the potato plants over the growing season that gives the greatest potential for a predictive modeling approach. In our study, data collection from just 4 flights over the growing season allowed us not only to identify the maturity of the different varieties, but also to better understand the canopy development of those varieties. Canopy architecture impacts light interception, water uptake and transpiration, important factors for carbon acquisition and allocation (Haverkort et al., 1991; Burgess et al., 2017; Tang et al., 2019). These represent some of the most valuable traits that breeders need to focus on for breeding improved crop varieties that are well adapted for meeting the challenges posed by climate change. Hence, use of the crop canopy assessment techniques described here will help to determine optimal plant architecture or ideotypes for different breeding purposes (Da Silva et al., 2014; Obidiegwu et al., 2015; Burgess et al., 2017).

### Opportunities and Challenges

Many studies have explored the potential of structure from motion techniques in life sciences. In the field of agriculture, the focus has been on monocot crops, specifically wheat, whose development is usually assessed via height measurements (Bendig et al., 2013, 2014, 2015; Holman et al., 2016; Jin et al., 2017; Hassan et al., 2019). Wheat, like most cereals, has a relatively homogeneous height distribution of the canopy when compared to potato. Potatoes are also grown on ridges or ridged rows where soil background is distinguishable in most scenarios and are a bush like crop in which ground cover is recognized as one of the main methods to evaluate growth. The pipeline developed in this work combines vertical growth with canopy cover data, as potato grows in a great variety of canopy shapes and structures that will be hard to capture with only a 2-dimensional parameter such as height or ground cover. This pipeline and the difference model creation allows us to capture the entire canopy distribution at ∼1 cm resolution and determine canopy cover and volume at different height levels during crop growth and development. That said, we want to highlight that structure from motion is one of the available techniques which can be used for obtaining surface information of crop canopies non-destructively. There are other approaches such as terrestrial laser scanning, laser triangulation, time of flight etc., which allow higher point cloud resolution (depending upon sensor and platform) and hence 3D sensing for plant phenotyping (Paulus, 2019).

## Conclusion

The paper highlights the application of existing tools for processing point cloud data obtained from UAV imaging for practical and accurate phenotyping of canopy architecture traits (plant height, canopy cover and volume) in potato which can be replicated in other bush type crops. In particular, the approach allows the consistent monitoring of canopy traits, which will facilitate the creation of accurate individual growth profiles for new and existing varieties. These profiles will enhance all future studies that assess not only varietal variability, but also its interaction with environmental factors (e.g., drought, temperature stress) and agriculture management practices (e.g., fertilization, tillage and crop rotation), thus supplying valuable environmental interaction data to help alleviate one of the current bottlenecks in genotype-phenotype association studies (Elias et al., 2016).

Using the newly developed and low-cost techniques, farmers could use the information from temporal monitoring of canopy size characteristics to identify key indicators of canopy age, health and development. For example, identification of early senescence (a potential indicator of stress), drooping due to stress or disease, or canopy lodging due to inadequate stem strength or maturity, thus facilitating the prediction of disease occurrence and spread. Identification of the current stage of the crop life cycle based on the detailed crop and variety profiles, in combination with other datasets, would allow farmers to determine the optimal time for harvesting based on varietal variation. These examples illustrate how a better understanding of the time course of crop development can inform important decisions and hence improve agricultural management practices.

## Data Availability Statement

The raw data supporting the conclusions of this article will be made available by the authors, without undue reservation, to any qualified researcher.

## Author Contributions

NB and AP conceived the project and designed the study. FdJ, JS, and AP performed the experiments, data collection, performed image data analysis, and drafted the manuscript. FdJ, NB, GB, LC, and AP participated in the experiments and statistical data analysis. NB, GB, and LC contributed with editing and revisions. All authors contributed to the article and approved the submitted version.

## Conflict of Interest

JS was employed by company Survey Solutions Scotland. The remaining authors declare that the research was conducted in the absence of any commercial or financial relationships that could be construed as a potential conflict of interest.
