# Supplementary material for: Development and Validation of Methodology for Estimating Potato Canopy Structure for Field Crop Phenotyping and Improved Breeding
Source: Front Plant Sci. 2021 Feb 10;12:612843. doi: 10.3389/fpls.2021.612843 (PMC7902928; doi:10.3389/fpls.2021.612843)
Supplement: Supplementary file 1 [file Data_Sheet_1.docx]

**Supplementary Material**


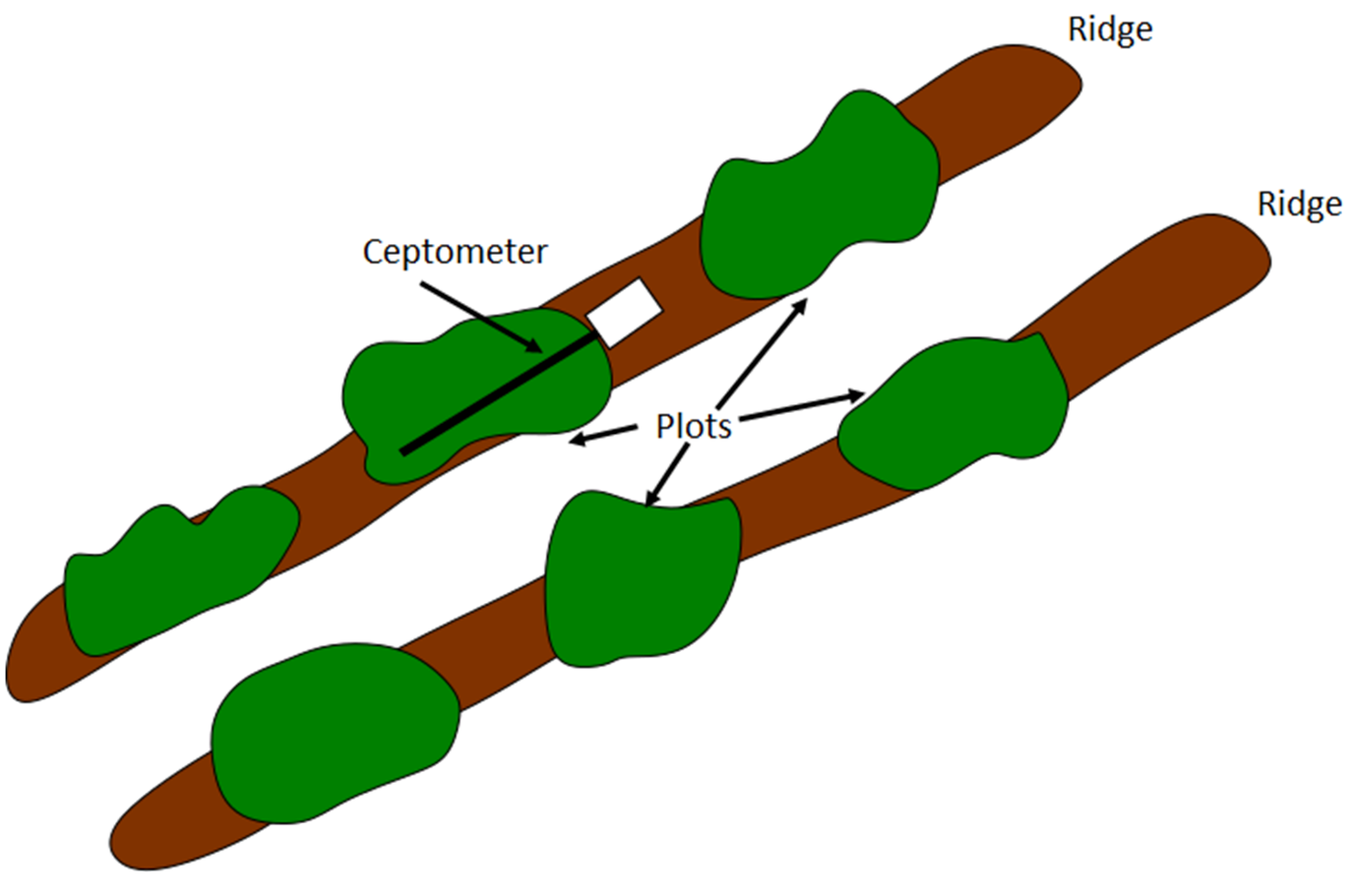


**Supplementary Figure S1**. Representation of the positioning of the ceptometer in relation to the canopy and ridges for LAI measurements. The crop canopy is shown in green and in brown is the outline of the ridges.


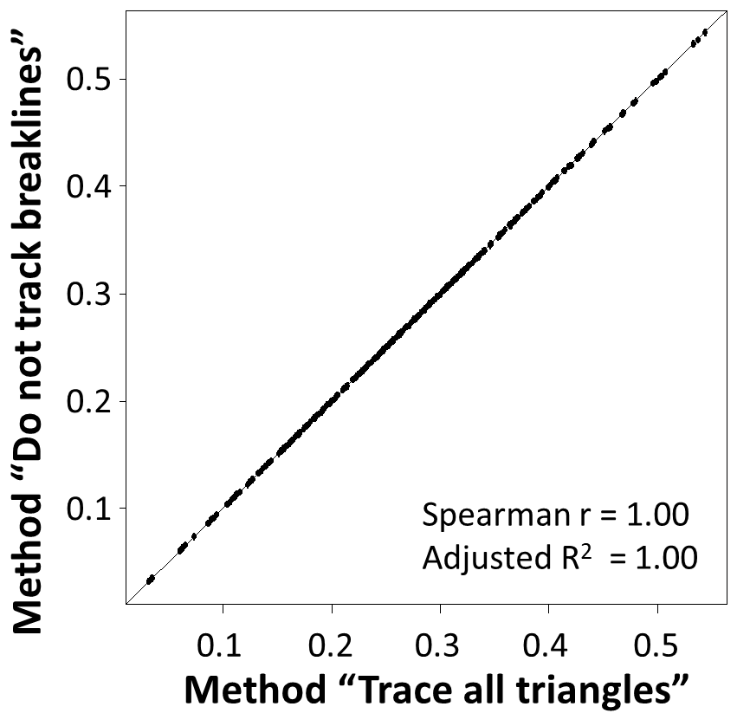


**Supplementary Figure S2**. Comparison of computed canopy volume (m^3^) using two difference model construction methods (“trace all triangles” and “do not track breaklines”). Spearman’s rank correlation r= 1.00, adjusted R^2^=1.00, p-value <0.01, n=300.
